# Circulating Endothelial Cells and Chronic Kidney Disease

**DOI:** 10.1155/2014/364738

**Published:** 2014-05-18

**Authors:** Kunying Zhang, Fang Yin, Lin Lin

**Affiliations:** Department of Nephrology, Weifang People's Hospital, No. 151 Guangwen Street, Kuiwen District, Weifang, Shandong 261041, China

## Abstract

Endothelial dysfunction may play a crucial role in initiation of the pathogenesis of vascular disease and atherosclerosis. The identification and quantification of circulating endothelial cells (CEC) have been developed as a novel marker of endothelial function. We describe, in great detail, mechanisms of endothelial dysfunction and CEC detachment. We also review the relationship between numbers of CEC and disease severity and response to treatment. In addition, we describe the possible clinical use of CEC in chronic kidney disease (CKD) and kidney transplantation. In summary, CEC have been developed as a novel approach to assess the endothelial damage. Measurement of the CEC level would provide an important diagnostic and prognostic value on the endothelium status and the long-term outcome of vascular dysfunction.

## 1. Introduction

Atherosclerosis is well known to be advanced in patients with chronic kidney disease (CKD). Cardiovascular disease (CVD) is one of the major causes of morbidity and mortality in this population, 10 to 20 times higher than in general population [1]. Association between CKD and cardiovascular complications is linked to a number of factors including traditional risk factors, such as age, gender, obesity, hypertension, hyperlipidemia, and nontraditional risk factors typical of CKD like uremic toxins, proteinuria, inflammation, alterations of mineral metabolism, and increased oxidative stress [2–4].

Recent evidence demonstrated that endothelial dysfunction may play a crucial role in initiation of atherosclerosis [5] and is, in general, the result of a series of interacting cardiovascular risk factors [6]. Endothelial dysfunction due to various factors results in increased monocyte infiltration and their differentiation into macrophages, which take up modified cholesterol-rich lipoproteins to form “foam cells” [7]. This may lead to the formation of atherosclerotic lesions.

Endothelial damage can be assessed in many ways [8–11]. Circulating endothelial cells (CEC) have been recognized as a potential marker of endothelial damage in a variety of vascular disorders during the last decade [12–15].

In this review we cover the possible contents of (1) introduction, (2) endothelial dysfunction in association with chronic kidney disease, (3) circulating endothelial cells, (4) measurement of circulating endothelial cells, (5) circulating endothelial cells—as a biomarker of endothelial dysfunction, (6) potential value of circulating endothelial cells in chronic kidney disease, (7) circulating endothelial cells in transplantation, and (8) conclusions.

## 2. Endothelial Dysfunction in Association with Chronic Kidney Disease

The endothelium, the largest organ in the body, comprises more than 10^13^ endothelial cells [16] and serves as a barrier separating the blood from the underlying tissue. The endothelium is essential for vascular haemostasis by secreting a number of vasoactive substances.

Endothelial dysfunction is an early event in arteriosclerosis and is observed even as early as in stage 1 CKD patients [17]. Increasing documents indicate that prolonged exposure to risk factors, such as inflammation and oxidative stress chronically present in CKD patients, may alter the normal homeostatic properties of the endothelium and active endothelial cells. As a consequence, the injury not only increases the adhesiveness of the endothelium to leukocytes or platelets, as well as its permeability, but also induces the endothelium to have procoagulant state and to form cytokines, vasoactive molecules, and growth factors [5, 18].

Among the insults to the vessel wall in CKD patients, uremia is regarded to be an elicitor of endothelial dysfunction. In uremic environment, there is an impairment of vascular endothelial nitric oxide synthase activity, and an increase in endothelial adhesion molecules such as von Willebrand factor, thrombomodulin, and circulating endothelial microparticles, which are stimulated by different uremic toxins [19–25]. Evidences obtained from the association between measurements of endothelial dysfunction and cardiovascular disease outcomes in uremic patients showed that impaired endothelial function may be an important mediator of cardiovascular disease risk in patients with end stage renal disease [26].

Vascular calcification is common in patients with chronic kidney disease as a result of inflammation, uraemia, and mineral metabolic disturbance. In disorders with high activation of bone morphogenetic protein, the endothelium is a source of osteoprogenitor cells in vascular calcification [27]. At the same time, vascular calcification may be one of the important mechanisms contributed to endothelial dysfunction. A recent study showed that vascular calcification was closely related to cardiovascular events [28]. This may be explained by a decrease in arterial compliance due to uremic vascular calcification [29].

## 3. Circulating Endothelial Cells

Circulating endothelial cells, a subpopulation of endothelial cells, are mature endothelial cells thought to originate from blood vessel walls and are released into the circulation in response to endothelial damage [30, 31]. Like tumour cells, CEC belong to the rare nonhematopoietic cells family present at a very low frequency, about 0–12 cells/mL blood in peripheral blood in the normal population [13, 30].

Bouvier and Hladovec were the first to describe the presence of CEC of possible endothelial origin in blood [32, 33]. These findings were subsequently confirmed both in animals such as influence on cerebral infarction [34] and in human being such as acute myocardial infarction [33], hypertension [35], and ANCA-associated small-vessel vasculitis [13].

Increased numbers of CEC detached from vessel wall due to a variety of factors related to endothelial damage, such as oxidative stress, infectious agents, cytokines, proteases [36], antiendothelial cell antibodies [37], and disturbed flow-induced p53 and ERK5 SUMOylation [38]. It is not known clearly whether CEC are proinflammatory [39]. In a cohort of granulomatosis with polyangiitis (GPA) patients with vasculitis and kidney involvement, the authors found that circulating inflammatory endothelial cells, a kind of endothelial cells that are detached from the site of inflammation and are released into the peripheral blood, could release increased inducible nitric oxide synthase and neutrophil-activating chemokines such as epithelial neutrophil-activating peptide-78, growth-related oncogene-*α*, macrophage inflammatory protein-1*α*, and IL-8 and induced elevated neutrophil migration. They concluded that these cells might be an additional inflammatory cell type that may play an important role in the pathogenesis of GPA [40].

The turnover time of the normal tissue endothelium is estimated to be 47 to 23000 days [41], and endothelial proliferation seems to be clustered at the sites of vascular branching [42]. Little is known about the clearance of CEC. A previous study has reported that the level of CEC may take as long as 7 days before returning to baseline after angioplasty [43]. However, clearance rates have not been examined directly.

## 4. Measurement of Circulating Endothelial Cells

Circulating endothelial cells are identified by the presence of endothelial cell specific/selective antigens [44]. A number of antigens have been used to identify cells of endothelial origin such as CD146, CD141, CD106, CD105, CD62e, CD54, CD31, and tissue factor [44–46]. In recent years, CD146 is the most popular choice for CEC identification, which is mainly expressed at the endothelial junction where it plays an important role in the control of intercellular cohesion, permeability, and signalization [47, 48]. However, CD146 is not restricted to the endothelium and is expressed by other cells such as T cells [49] and stromal cells [50], it can not be used alone to identify the CEC, and combination with some other biomarkers or additional techniques is needed.

To date, standardization of methods for quantification of CEC has not been well established. Improved detection of these rare cells has been achieved by combining cell enrichment techniques with labeling of CEC with certain selective surface antigens. Most studies isolate CEC either by immunomagnetic separation or by flow cytometry from blood samples [15, 51–54]. Immunomagnetic separation is regarded as the superior method for CEC quantification in a recent consensus guideline [51]. It commonly uses immunomagnetic beads coated with antiendothelial antibodies mixed with venous blood. Bound CEC are retained by magnet; unbound CEC are washed out by buffer. CEC are identified according to size and number of beads bound [30, 55]. Flow cytometry is another attractive method to quantify CEC [40, 56, 57]. It tends to use a combination of different surface antigens such as CD146, CD45, and CD31 to detect the endothelial cells [58]. However, both techniques have their advantages and disadvantages [59]. Nonspecific binding is one of the most important reasons to make CEC quantification more complicated. Hence, more attention to the best approach of CEC measurement is required.

## 5. Circulating Endothelial Cells: As a Biomarker of Endothelial Dysfunction

Endothelial dysfunction can be assessed in several ways, for example, by physiological techniques as flow mediated dilatation [8] and by the measurement of certain markers as endothelin [9], microalbuminuria [10, 60], soluble E selectin, cell adhesion molecules, and von Willebrand factor [6, 11] in the peripheral blood.

More recently, emerging evidence suggest that CEC have been developed as a sensitive and specific marker for assessing endothelial damage in both renal [15, 61] and nonrenal [62] patient groups and can be closely related to other indices of endothelial dysfunction, such as plasma von Willebrand factor (vWf), tissue factor, IL-6, and impaired FMD [62, 63]. Moreover, unlike biochemical indicators, which may not be endothelium-specific, CEC originate directly from the endothelium and accordingly are better representatives of the endothelial status.

In certain disease conditions, CEC detached from affected vascular wall could provide useful information for studying vascular injury. Increased levels of CEC have been observed in several diseases with widespread vascular damage, as recently demonstrated in patients with sickle cell anemia [12, 64], infection with cytomegalovirus [65], ANCA-associated small-vessel vasculitis [13], systemic lupus erythematosus [66], Behcet's disease [67], acute coronary syndrome [68], acute myocardial infarction, unstable angina [69], and severe peripheral artery disease [12, 62, 69]. Moreover, elevated levels of CEC showed a strong association with disease severity and outcome. CEC levels are higher in patients with acute illness than those in recovery phase or in clinical remission of the disease [68, 69]. Several lines of evidence suggest that treatment of the underlying disease may restore endothelial function and decline the CEC levels. For example, In Mediterranean spotted fever, a strong correlation between CEC numbers and disease severity was found in patients with malignant forms of the disease who developed thrombotic complications and sometimes required anticoagulant therapy, while the cell numbers declined progressively during treatment and recovery [70]. Such relationship has been found in the model of the disseminated human cytomegalovirus (CMV) infection [71]. Makin et al. demonstrated that patients had higher CEC level in critical limb ischaemia than in intermittent claudication [62]. Mutin et al. [69] found that CEC numbers increased in patients with acute myocardial infarction compared to patients with angina [69].

## 6. Potential Value of Circulating Endothelial Cells in CKD

Increased CEC numbers are present in various forms of kidney diseases. As a marker of endothelial damage, the measurement of CEC could provide important value in the clinical setting.

In maintenance hemodialysis (MHD) patients, Koç et al. reported that the number of CEC was increased compared with healthy controls, and even higher level of prehemodialysis CEC was observed in patients with active atherosclerotic cardiovascular disease (ACVD) compared with patients with stable ACVD and no ACVD [15]. In the subsequent study, they found that the increase of CEC level was associated with the high prevalence of cardiovascular and vascular events and was independent of other known markers of inflammation or endothelial dysfunction [72]. In our recent study, we found that the level of CEC was higher in MHD patients than in healthy subjects, too. Furthermore, we found that predialysis CEC level positively correlated with increased intima-media thickness of common carotid artery (CCA-IMT) even after adjusting the confounding effects. An interesting finding in this study was that division of patients into three subgroups based on CCA-IMT showed that greater thickness of CCA-IMT correlated with greater number of CEC [73]. These evidences may suggest that CEC could be a potential marker of the state of the endothelium and could be a useful indicator in predicting cardiovascular risk in maintenance hemodialysis patients.

In systemic autoimmune diseases, such as systemic lupus erythematosus (SLE) and ANCA-associated vasculitis, endothelial cells are targets of antibody or immune complex-mediated injury. Increased numbers of CEC also have a good relationship with the severity of these diseases. In systemic lupus erythematosus, patients with active disease expressed higher level of CEC in peripheral blood compared with patients with inactive disease or healthy subjects [66]. Yao et al. [74] demonstrated that, in SLE patients with renal vascular lesions, the numbers of CEC were significantly higher than those without vascular lesions, and, in all lupus nephritis patients with vascular lesions, CEC level of the patients with thrombotic microangiopathy (TMA) significantly increased compared with those without TMA. The similar finding was observed in pure TMA [75]. Woywodt et al. found high level of CEC in patients with active ANCA-associated small vessel vasculitis, a paradigm of an endothelial disorder, whereas cell numbers declined progressively during the successful therapy [13].

Data in glomerular diseases are limited. Even in the normal aging process, it has been shown that the loss of glomerular endothelium is associated with progressive renal impairment. Futrakul et al. demonstrated that enhanced CEC level, elevated transforming growth factor beta, and depleted vascular endothelial growth factor were observed in patients with focal segmental glomerulosclerosis (FSGS), and they presumed that the increased endothelial cell loss may be due to the elevated transforming growth factor beta, which can induce apoptosis of podocyte as well as tubular epithelium [76]. But elevated CEC numbers were not found in patients with glomerular disease in another relatively smaller numbers of patients [13].

In CKD patients, increased CEC level was found in patients with moderately to severely impaired renal function (creatinine clearance less than 60 mL/min/1.73 m^2^) [77], even in patients with concomitant mild renal dysfunction [15]. Rodríguez-Ayala et al. reported that increased CEC expressing MHC class-I-related chain A (MICA) and decreased endothelial progenitor cells (EPC) expressing Tie-2 or VEGFR-2 were found in a group of CKD patients with advanced renal impairment, which suggested a marked imbalance between CEC and EPC (a surrogate marker for vascular repair) in these patients [78].

## 7. Clinical Value of Circulating Endothelial Cells in Transplantation

In patients with kidney transplantation, the risk of cardiovascular disease is higher than in general population, although cardiovascular mortality is lower compared with those receiving dialysis [79].

Recently, the level of CEC has been identified as a useful marker of endothelial damage and potential vascular rejection in renal transplant recipients. More and more studies showed that CEC numbers are elevated in patients after kidney transplantation [14, 61, 80]. This discrepancy could be explained by cytomegalovirus infection, allograft rejection, and use of calcineurin inhibitors such as cyclosporine [14]. Woywodt et al. demonstrated that patients with acute vascular rejection had the highest cell numbers compared with other patients [61]. Mohamed et al. found that the cells seemed to be derived from the graft itself [81]. Therefore, kidney transplantation may have a remarkable effect on endothelial damage. Interestingly, CEC level is significantly decreased in patients one year after kidney transplantation [82].

## 8. Conclusions

Taken together, the above accumulative evidence indicates that endothelial dysfunction contributes to the development of cardiovascular and renal disease. Circulating endothelial cells have been developed as a novel approach to assess the endothelial damage in various disorders including chronic kidney disease. Evaluating CEC directly detached from vessel wall may provide direct insights on the status of the endothelium, as well as providing mechanisms underlying endothelial dysfunction. Increased CEC level is closely related to clinical development of vascular disease in patients with chronic kidney disease. Treatment of the underlying disease may recover endothelial function and decreased the CEC levels. In particular, CEC are easily obtainable from blood samples. So, measurement of the CEC level would provide an important diagnostic and prognostic value on the endothelium status and long-term outcome of vascular dysfunction.
